# A Unique Case of a Child with Two Rare Hereditary Diseases: Familial Dilated Cardiomyopathy and Arterial Calcification

**DOI:** 10.3390/ijms26125900

**Published:** 2025-06-19

**Authors:** Yulia Burykina, Daria Chudakova, Olga Zharova, Elena Basargina, Irina Silnova, Natalia Sdvigova, Leila Gandaeva, Yulia Davydova, Valentina Kaverina, Ilya Zhanin, Alexander Pushkov, Andrey Fisenko, Kirill Savostyanov

**Affiliations:** 1 National Medical Research Center of Children’s Health of the Ministry, Health of the Russian Federation, 119991 Moscow, Russia; burykina.ius1@nczd.ru (Y.B.);; 2 N.F. Filatov Clinical Institute of Children’s Health, I.M. Sechenov First Moscow State Medical University of the Russian Ministry of Health (Sechenov University), 119992 Moscow, Russia

**Keywords:** next-generation sequencing, dilated cardiomyopathy, arterial calcification

## Abstract

Here, we present a unique case of the combination of two rare hereditary diseases—a familial form of dilated cardiomyopathy (DCM) and arterial calcification (AC)—in a 10-month-old boy. DCM was caused by a novel pathogenic nucleotide variant (NV) *c.542G>T* in the *MYH7* gene, and AC was caused by biallelic nucleotide variants *c.3421C>T* and *c.4015C>T* in the *ABCC6* gene. NVs were identified by the next-generation sequencing (NGS) of a broad panel of 404 genes potentially involved in cardiovascular disorders and subsequently validated by Sanger sequencing in the proband and his parents. Cardiologic examinations confirmed the familial nature of cardiomyopathy and the pathogenicity of variant *c.542G>T* in *MYH7* gene. This case highlights the clinical utility of NGS in identifying complex co-existing hereditary conditions and emphasizes the need for the comprehensive genetic testing of patients with atypical clinical presentations.

## 1. Introduction

Cardiovascular diseases (CVDs) are a large group of vasculature and cardiac disorders; some of them have strong genetic components, being either monogenic conditions or disorders resulting from a complex interplay of multiple genetic factors. Cardiomyopathies (CM) are disorders of heart muscle from the group of CVDs, classified as dilated (DCM), hypertrophic (HCM), restrictive (RCM) and arrhythmogenic right ventricular dysplasia (ARVD/CM). DCM is characterized by the enlargement of the left ventricle (LV) of the heart (an end-diastolic size of the LV exceeding Z-scores of 2) and a systolic dysfunction (an ejection fraction of the LV below 55% according to Simpson’s method), which cannot be solely attributed to abnormal loading conditions such as hypertension or coronary artery disease.

As per the latest data from the European Society of Cardiology (ESC) from 2023, the prevalence of the DCM phenotype in children over the age of 1 year is approximately 26 cases per 100,000 individuals, whereas in infancy it ranges from 38 to 46 cases per 100,000.

Currently, primary (genetically determined) and secondary (arising from non-genetic causes) DCM are distinguished. Hereditary forms of DCM account for 20 to 50% of all cases [1]. They result from pathogenic sequence variants (PSVs) in a wide array of over 50 genes, mainly encoding proteins crucial for cardiac muscle contraction and structural integrity. Predominantly, DCM has an autosomal dominant pattern of inheritance, but also can be inherited in an autosomal recessive or X-linked manner. The common genetic causes of pediatric DCM are PSVs in *TPM1* (gene encoding tropomyosin), *TNNT2* (gene encoding cardiac troponin T), and other so-called sarcomeric genes [2,3]. Growing evidence shows that PSVs in *MYH7*—the gene encoding the beta heavy chain of cardiac myosin—are also among the causes of hereditary DCM, though they are not the predominant cause [4].

DCM is characterized by a progressive course, leading to chronic heart failure (CHF), and can cause various heart rhythm disturbances, as well as sudden cardiac death (SCD). During the clinical examination of an individual with suspected CM, it is important to focus on the features of the phenotype and extracardiac symptoms, which can be indicative of specific syndromes that include CM in their symptom complex. Examples of such syndromes are Barth [5], Alström [6], and 1p36 deletion syndromes [7]; Danon disease; RASopathies [8,9]; and Fabry disease [10], etc.

Various approaches are currently used to establish correct DCM diagnosis, such as echocardiography (EchoCG), Holter monitoring of ECG (HM-ECG), CT coronary and aortography, and magnetic resonance imaging (MRI) of the heart. Genetic testing is essential for establishing correct diagnosis and informed decisions regarding the use of implantable defibrillators to prevent SCD in case of hereditary DCM. Given that many genes are known to be involved in pathogenesis of DCM, the best tool for genetic diagnostic of DCM is targeted next-generation sequencing (NGS), allowing for a fast and cost-efficient analysis of a broad panel of genes.

Notably, the presence of PSV causatives of DCM does not exclude the possibility that the patient might have other hereditary conditions also affecting cardiovascular systems and hence resulting in a complex phenotype which requires personalized therapy adjustment. For example, hereditary disorders of cardiovascular calcification, such as arterial calcification of infancy, might result in hypertension [10], which, in turn, impacts the progression of DCM. Moreover, in small, isolated populations or societies with traditions of consanguineous marriage, the risk of genetic disorders (and hence the chances that the individual might have two or more rare genetic disorders) might be elevated due to increased homozygosity and founder effects [11].

Recently, there has been a marked rise in publications reporting coexistence of two or more rare genetic disorders within individual patients and highlighting the need for comprehensive genetic evaluation in clinical practice. For example, a study by Ebbinghaus et al. describes the dual presence of two rare cardiac disorders in single individuals: the co-existence of cardiac sarcoidosis and inherited cardiomyopathy, with a phenotypic overlap [12]. Another recent study reports a family with co-occurrence of three inherited rare diseases including cardiomyopathy and multiple extracardiac abnormalities [13]. Similarly to our report, a unique case of two genetic disorders (Kabuki syndrome and Charcot–Marie–Tooth disease) in one patient has been described recently, including the identification of a novel genetic variant [14]. Furthermore, a remarkable triple genetic diagnosis—mosaic Jacobs syndrome, X-linked chondrodysplasia punctata, and *MECP2*-related disorder—was established in one patient using rapid whole-genome sequencing (rWGS) [15]. A retrospective study of a large cohort of 1487 pediatric patients demonstrated that cases with more than one genetic disorder in one patient are more common than previously estimated, especially in patients with complex or multisystem phenotypes [16]. Therefore, it has been suggested that, in genetic diagnostics, the possibility of dual or multiple hereditary conditions should always be taken into account when evaluating patients with atypical clinical presentations [17]. Identifying such cases via comprehensive genetic analysis clarifies the etiology of overlapping symptoms and guides tailored surveillance and treatment strategies. For example, a very recent case report describes a 10-year-old girl diagnosed with two rare hereditary conditions—Wilson’s disease (WD) and Alexander’s disease (AxD) based on the whole-exome sequencing (WES) findings [18]. This dual diagnosis explained complex clinical presentation with atypical findings. Establishing proper diagnosis in turn enabled personalized multidisciplinary treatment of the patient.

This report presents a unique case of a child with familial DCM, caused by a pathogenic nucleotide variant in *MYH7*, and AC, caused by pathogenic nucleotide variants in *ABCC6* gene, identified by targeted next-generation sequencing (NGS) of a panel of 404 genes, which is a perfect example of such a scenario. To the best of our knowledge this is the first such case report in the literature worldwide and the first report describing variant *c.542G>T* (corresponding to amino acid variant p.G181V) in gene *MYH7*.

## 2. Materials and Methods

### Genetic Analysis

The peripheral blood was dried on a filter paper (whatman carta-filter No. 903) and genomic DNA was isolated from dried blood spots using “MagPure Universal DNA Kit” (Magen, Guangzhou, China), following manufacturer’s recommendations. Following elution, the quantity of DNA was assessed by a fluorometer “Qubit 3.0” (Invitrogen, Waltham, MA, USA) using Qubit dsDNA HS Assay Kit. The target regions of the custom panel of 404 genes known to be involved in cardiovascular diseases were analyzed by NGS as previously described, with some modifications. NGS library preparation was performed using KAPA Hyper plus Kit (Roche, Indianapolis, IN, USA), following manufacturer’s instructions. DNA (~150 ng) was fragmented for 15 min to achieve a fragment length of approximately 350 bp. Kapa custom panel technology (Roche, USA) was used for the target enrichment. Massive parallel sequencing was performed on the NextSeq 2000 platform (Illumina, San Diego, CA, USA) with P3 chemistry (300 cycles, paired-end reads). On average, each sequencing run yielded 2.5 billion pass-filtered reads, with 88% of these reads achieving a Phred quality score above Q30, indicating an error rate of less than 1 in 1000 bases. Bioinformatic analysis was performed following the GATK Best Practices guidelines (https://gatk.broadinstitute.org/, accessed on 1 June 2025). The workflow included the following steps: (1) Raw reads were trimmed using Trimmomatic (version 0.39); (2) reads were aligned to the GRCh38p14 reference genome using bwa-mem2 (version 2.2.1); (3) duplicate reads were marked with Picard tools v3.2, followed by base quality score recalibration (BQSR); (4) variant calling for SNPs and indels was performed using GATK HaplotypeCaller (version 4.6.1); and (5) gene annotation was performed using an in-house script to annotate variants present in ClinVar, OMIM, and HGMD.

In silico tools SIFT v6.2.1, PolyPhen HDIV v2.2.3, PolyPhen HVAR v2.2.3, Mutation Taster 2021, FATHMM v2.3, CADD13 v1.6, DANN v1.0, M-CAP v1.4 and REVEL v1.0, as well as Alamut v.1.7.2 with the built-in software modules were used for the prediction of the pathogenicity of the missense sequence variants. The guidelines of the American College of Medical Genetics and Genomics (ACMG) manual were used for the variants interpretation.

NGS results were validated by Sanger sequencing. Family segregation analysis of nucleotide variants detected in the proband was also performed by Sanger sequencing. Sanger sequencing was performed via the BigDye^®^ Terminator v3.1 Cycle Sequencing Kit (Thermo Fisher Scientific, Waltham, MA, USA) in accordance with the manufacturer’s protocols and guidelines. Amplification was performed on Bio-Rad T100 (Bio-Rad, Hercules, CA, USA) and ProFlex (Thermo Fisher Scientific, USA) thermocyclers. Capillary electrophoresis was performed on ABI 3500XL automated DNA sequencer (Thermo Fisher Scientific, USA). The obtained sequences were compared with RefSeqGene reference sequences from the National Center for Biotechnology Information database.

## 3. Results

Here, report a unique case of a child presenting with familial cardiomyopathy and idiopathic arterial calcification, diagnosed with the aid of NGS.

### Case Presentation

A 10-month-old boy was hospitalized in the cardiology department of the National Medical Research Center of Children’s Health of the Ministry of Health of the Russian Federation with a referral diagnosis of DCM. Anamnesis: the child was born to unrelated parents who considered themselves healthy, from the first pregnancy without peculiarities to first-term physiologic delivery. Birth weight: 3200 g; body length: 52 cm; Apgar score: 8/8 points. Past medical history was as follows: EchoCG at 1 month of age revealed no pathology. Until 7 months of age, the patient grew and developed according to his age. At 7 months, the parents noted decreased appetite, poor weight gain, delayed motor development and dyspnea. Due to the suspicion of congenital heart disease (CHD), he was examined. EchoCG revealed pronounced dilatation of heart cavities and increased trabecularity of LV myocardium. Taking into account his age and echoCG data, an anomalous origin of the left coronary artery from the pulmonary artery was suspected. He was referred to a cardiac surgery hospital to exclude CHD. EchoCG revealed marked dilatation of the left heart chambers (LV 47/41 mm, spherical shape, left atrium (LA) 31 mm), moderate atrioventricular valve insufficiency, increased trabecularity of LV myocardium, decreased ejection fraction (EF) up to 27.5%, pulmonary hypertension up to 68 mm Hg, no evidence of CHD. Electrocardiography (ECG) revealed sinus tachycardia with heart rate (HR) up to 157 per min. Radiography detected cardiomegaly with increased cardiothoracic index (CTI) 70%. Laboratory analysis showed a marked increase in the N-terminal fragment of brain natriuretic peptide (NTproBNP) up to 7352.7 pg/mL.

The patient was transferred to the cardiology department of the Federal State Institution “Children’s Health Center” of the Ministry of Health of Russia for further examination and decision on management tactics.

At the initial hospitalization, marked symptoms of heart failure (tachycardia up to 152/min, tachypnea with respiratory rate (RR) up to 60/min with retractions and grunting, hepatomegaly +3 cm below the edge of the rib arch) and muscular hypotonia were noted. On examination, blood pressure (BP) was normal for a non-verticalized child—84/34 mmHg in the arms and 76/40 mmHg in the legs. Physical development was assessed as an average, which was disharmonious due to a body weight deficiency of up to 25%: weight was 6.2 kg, body length 71 cm, Z-score height/age −1.08, Z-score weight/age −3.53, and Z-score weight/height −4.14. A preliminary diagnosis was established: DCM increased the trabecularity of the left ventricular myocardium, atrioventricular valve insufficiency, CHF 2B-A stage FC III according to Ross, and nutritional deficiency.

**Figure 1:**
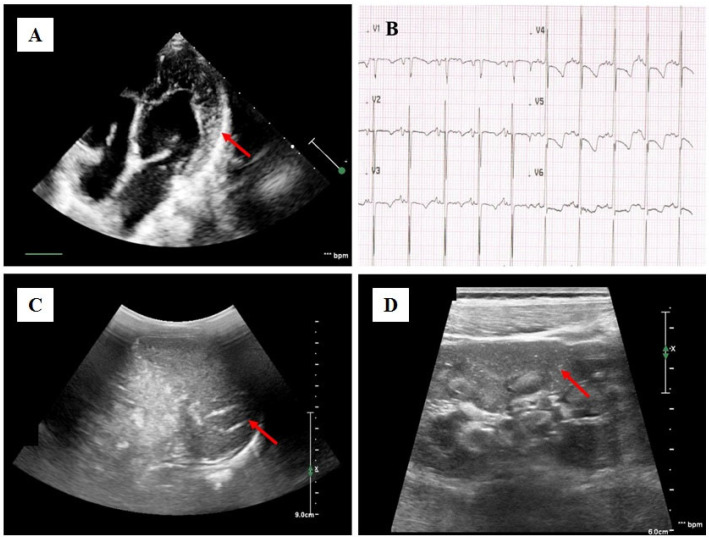
Clinical characteristics of the patient. Transthoracic echocardiography of the patient at the initial examination in our center, apical four-chamber position. The red arrow is pointing at deep trabeculae and intertrabecular recesses in the inferior, lateral, anterior walls, middle and apical portions of the septum and apex of the left ventricle (**A**). Fragment of electrocardiogram (thoracic leads) of the patient at the initial examination in our center: sinus rhythm, signs of atrial overload (P wave amplitude up to 2.5–3 mm), slowing of intraventricular conduction with QRS up to 85 ms, signs of repolarization disorder in the form of ST segment depression in leads V4–V6 up to 2 mm with negative T waves (**B**). Ultrasound examination of the abdomen. The red arrow is pointing at the multiple intraorgan arteries with thickened walls of increased echogenicity, which are detected in the structure of the spleen (**C**). The red arrow is pointing at multiple point hyperechogenic inclusions—intrarenal arteries with increased echogenicity of the walls are detected in the structure of the renal cortical layer (**D**).

Dynamics and outcomes were as follows. According to EchoCG: marked dilatation of the left heart chambers (left ventricular end-diastolic diameter (LV EDD 39 mm, Z-score +5, spherical shape with sphericity index (SI) 0.75, enlarged left atrium (LA) 30 × 31 mm, Z-score +5.2), decreased contractility with EF 27% according to Teicholz, 23% according to Simpson due to diffuse hypokinesis, signs of noncompact myocardium (noncompact layer 16 mm, compact layer 2.5 mm) (Figure 1A), moderate mitral valve insufficiency, mild tricuspid valve insufficiency, type 1 diastolic dysfunction (relaxation disorder), and no evidence of pulmonary hypertension.

According to ECG data (Figure 1B) and daily ECG monitoring by Holter—sinus rhythm, slowing of intraventricular conduction (QRS up to 85 ms), signs of atrial overload (amplitude of the *p*-valve up to 2.5–3 mm), heart rhythm disturbances and rhythm pauses were not registered. During ultrasound examination, perivascular hyperechogenicity of the spleen and kidneys drew attention (Figure 1C,D). Data from laboratory tests showed a significant increase in NTproBNP of up to 18,495 pg/mL.

The child was consulted by a medical geneticist, and a molecular genetic study by high-throughput sequencing was recommended. NGS analysis revealed heterozygous NV chr14:23431858C>A (*c.542G>T*, NM_000257.4) in exon 7 of *MYH7* gene, corresponding to amino acid variant p.G181V. No other clinically significant nucleotide variants in this gene were identified.

The in silico tool SIFT v.6.2.1 predicts this variant as pathogenic with high confidence. Mutation Taster is a variant of uncertain pathogenicity, and FATHMM suggests moderate pathogenicity. DANN has uncertain pathogenicity, M-CAP has moderate pathogenicity, and REVEL strongly supports the pathogenicity of this variant (see Supplementary Table S1). The following ACMG criteria were used to classify this variant and justify its pathogenic/likely pathogenic status: PM1 (exonic hotspot; 14 pathogenic or likely pathogenic reported variants were found in a 109 bp region surrounding this variant in exon 7 within the region 23431760–23431869 without any missense benign variants), PM2 (absence or extremely low frequency of this variant in population databases), PM5 (novel amino acid substitution at the same residue as previously reported pathogenic variant), PP2 (Gnomad constraint of missense upper Z-score for gene is greater than 3), PP3 (aggregated score predicts a deleterious effect).

**Figure 2:**
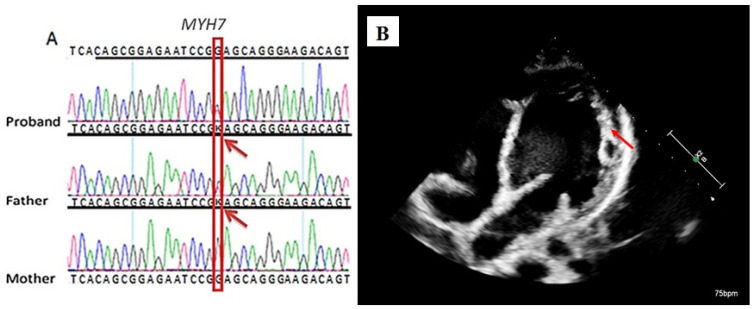
Familial nature of the cardiomyopathy in the studied case. Sanger sequencing validates the presence of chr14:23431858C>A, the *c.542G>T* variant of *MYH7* gene, in the proband and his father. K represents either G or T; nucleotide variants are indicated by the red arrow (**A**). Transthoracic echocardiography of the patient’s father at age 42. The red arrow is pointing at noncompact myocardium is visualized in the region of the apical and middle segments of the left ventricle (**B**).

Familial segregation analysis by Sanger sequencing showed that this variant was inherited from the proband’s father (Figure 2A), in whom at the age of 42 years, EchoCG revealed noncompact myocardium with impaired diastolic myocardial function and borderline systolic function, suggesting the diagnosis of noncompact myocardium of the left ventricle (Figure 2B).

The therapy for congestive heart failure (CHF) was adjusted: cardiac glycoside digoxin 10 mcg/kg/day, angiotensin-converting enzyme inhibitor captopril 2 mg/kg/day, beta-blocker carvedilol 1 mg/kg/day, aldosterone antagonist spironolactone 0.5 mg/kg/day, loop diuretic torsemide 0.5 mg/kg/day, and thromboembolism prevention with acetylsalicylic acid. A positive dynamic was noted during the observation period: dyspnea had stopped, and the liver size decreased to +0.5 cm.

Patients underwent a follow-up examination at the age of 1 year and 2 months. According to echocardiography, the sizes of the heart chambers decreased relative to height (LV EDD 33/25 mm, Z-score +2.6, LA 20 × 31 mm, Z-score +1.9), myocardial contractility improved (EF 50% according to Teicholz), diastolic function normalized, and regurgitation on the atrioventricular valves decreased. Noncompact myocardium had a ratio of 17:3 mm; according to ECG and HM-ECG, no cardiac arrhythmia or pauses were recorded; NTproBNP decreased to 499 pg/mL.

Objectively, the symptoms of heart failure were relieved. There was no tachycardia (HR 105 per min) or dyspnea (RR 25 per min), there was a tendency towards normalization of weight (7.6 kg) and height (75 cm), Z-score height/age −1.13, Z-score weight/age −2.65. However, for the first time, an increase in blood pressure to a maximum of 120/70 mm Hg was recorded. Thus, the calcium channel blocker amlodipine was prescribed, leading to a decrease in blood pressure to 90–100/60 mm Hg.

At the age of 2 years and 2 months, the patient was readmitted to hospital. The manifestations of CHF were clinically relieved: body weight deficit was relieved to −2.5 Z-score and elevated blood pressure persisted to 118/70 mm Hg. Laboratory tests showed a decrease in the NTproBNP level (112 pg/mL), echocardiography showed normalization of the heart chamber sizes (LV EDD 31/17 mm, Z-score +0.9, LA 22 × 30 mm, Z-score +1.8), and systolic function was normalized (EF 75% according to Teicholz, 61% according to Simpson).

**Figure 3:**
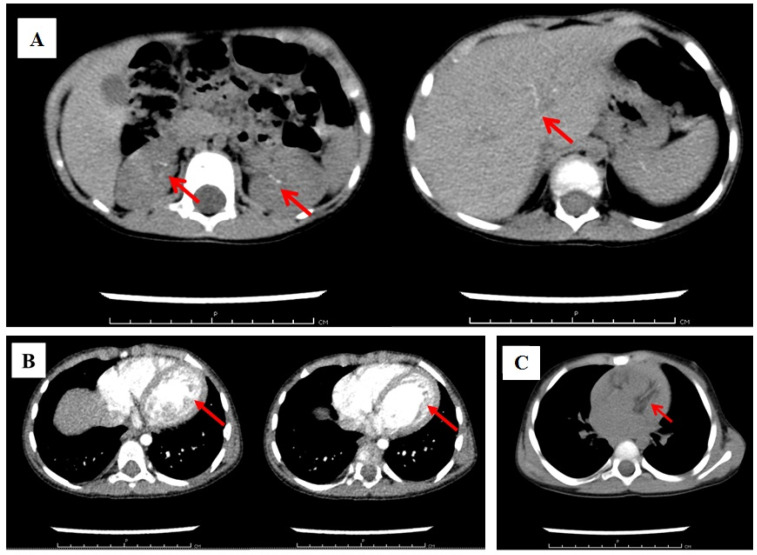
Results of multispiral computed tomography with intravenous contrast. Fragmentary calcification of hepatic, renal, and splenic arteries. The red arrow is pointing at the calcification (**A**); the red arrow is pointing at noncompact myocardium of the left ventricle and its dilation (**B**); the red arrow is pointing at calcification of the coronary arteries (**C**).

Taking into account the persistent arterial hypertension, as well as the previously identified calcification of the vessels of the spleen and kidneys based on the ultrasound examination of the abdominal organs, multislice computed tomography (MSCT) with intravenous contrast was performed (Figure 3).

Fragmentary calcifications of the branches of the hepatic, renal, splenic, and pancreatic arteries; aortic arch; coronary arteries; thymus without significant areas of vascular narrowing were detected. Noncompact myocardium of the left ventricle and its dilation were also visualized.

**Figure 4:**
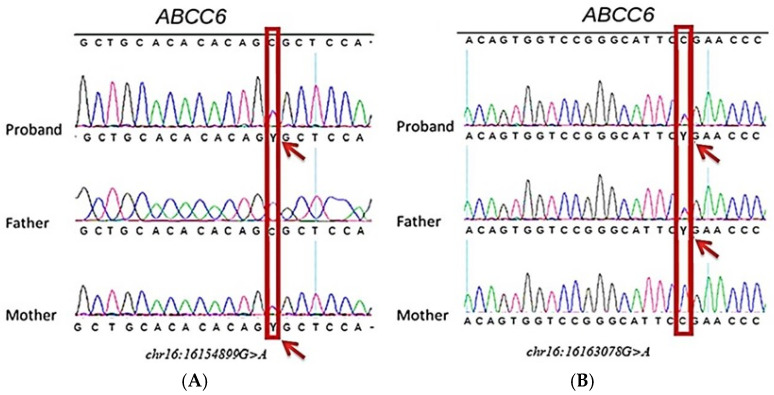
Results of *ABCC6* gene Sanger sequencing. The chr16:16154899G>A, *c.4015C>T* (**A**) and chr16:16163078G>A, *c.3421C>T* (**B**) were detected in the proband and his mother and father. Nucleotide variants are indicted by the red boxes/arrows, and Y represents either C or T.

Based on arterial hypertension and MSCT data, generalized arterial calcification was suspected, and bioinformatic reanalysis was recommended. Two NVs, chr16:16163078G>A, *c.3421C>T* (corresponding to amino acid variant p.R1141X) and chr16:16154899G>A, *c.4015C>T* (corresponding to amino acid variant p.R1339C) in the *ABCC6* gene exons 24 and 28, respectively, were detected by NGS. Both variants are known to be pathogenic. The results of NGS were confirmed by Sanger sequencing, revealing the presence of NVs *c.3421C>T* and *c.4015C>T* of the gene *ABCC6* in the proband (Figure 4).

**Figure 5:**
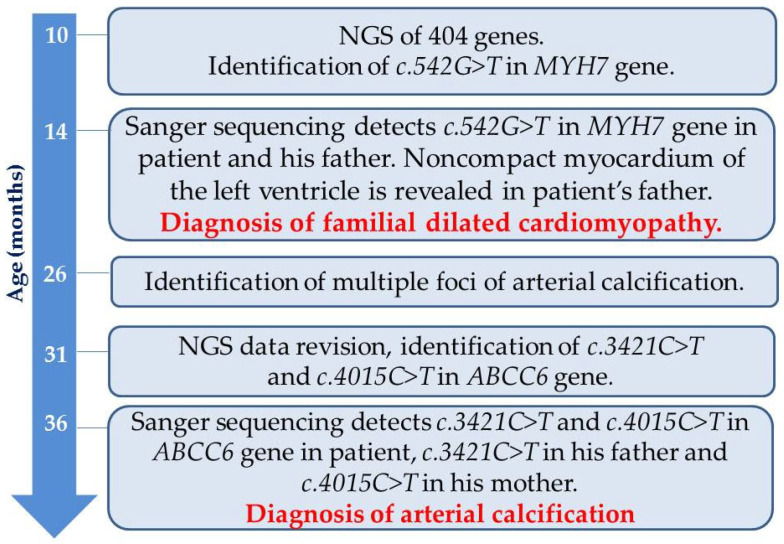
The timeline of the patient’s “diagnostic journey”. Definitive diagnosis points are highlighted in red.

Subsequent analysis of the proband’s parents identified *c.3421C>T* in the mother and *c.4015C>T* in the father. According to ultrasound data, no signs of calcification were detected in the father of the proband. Overall, the timeline of the patient’s “diagnostic journey” is summarized in Figure 5.

## 4. Discussion

Here, we present a unique case of co-existence in one patient of two rare genetic conditions affecting the cardiovascular system—DCM and AC—which results in a complex phenotype. The *MYH7* gene, located on chromosome 14q11.2, encodes beta-myosin heavy-chain protein MYH7, which is crucial for cardiac muscle contractility and a major myosin heavy chain protein in the left ventricular in adults. To the best of our knowledge, this report is the first to describe the *c.542G>T* variant in *MYH7*, and it has not been previously described in the control sample gnomAD, v4.1. Corresponding amino acid variant p.G181V is located in the ATP binding region, spanning residues 178–185 [19], which forms a part of ATP binding pocket of a globular head of myosin, critical both for ATP-hydrolysis and for actin–myosin interaction [20]. Based on its localization, this variant may impair these processes leading to inefficient contraction, although further functional studies are needed to test such assumptions.

Previously found amino acid variant p.R190T in MYH7 is also located in close proximity to the ATP binding site of MYH7 and is linked to familial HCM [21]; the nearby located variant p.I201T is associated with DCM [22].

Moreover, a recent study by Khan et al. describes variant c.541G>A p.G181R in *MYH7* (affecting the same amino-acid as in case of our variant p.G181V) found in pediatric patients with dilated cardiomyopathy with left ventricular noncompaction features (DCM-LVNC) and classified as pathogenic/likely pathogenic (P/LP) [23]. This variant affects part of the myosin, which is critical for its nucleotide-binding and motor functions, likely leading to altered sarcomere contraction and relaxation dynamics. It is a clinically actionable variant listed in ClinVar and Human Gene Mutation Database (HGMD) as disease-causing.

In the case of the p.G181R substitution, it introduces a bigger, positively charged amino-acid residue, whereas p.G181V introduces a nonpolar, hydrophobic residue. From the biochemist’s point of view, the latter change is less drastic compared to the former, thus it is reasonable to suggest that its impact on myosin function might be milder. The rod domain of MYH7 protein has an α-helical coiled–coil structure, where small nonpolar aminoacid residues (such as glycine) allow for tight packing and flexibility [24]. Thus, loss of glycine in case of p.G181R or p.G181V substitution might decrease protein flexibility by destabilizing the coiled–coil structure, hence impairing myosin filament assembly and muscle function.

It appears that most PSVs in *MYH7* are located in the gene region encoding globular head of myosin [15,25], thus it is not surprising that NV *c.542G>T,* which is also located in this region, was found in our study in pediatric patient with DCM.

The targeted NGS of the 404 genes involved in cardiovascular disorders also identified two NVs in the *ABCC6* gene in this patient. The *ABCC6* gene, located on chromosome 16p13.1, encodes protein *ABCC6* (also known as multi-drug resistance-associated protein-6, MRP6), a member of the superfamily of ATP binding cassette (ABC) transmembrane transporters. PSVs in *ABCC6* can be implicated in pseudoxanthoma elasticum (PXE), a genetic disorder characterized by calcifications in many organs/systems, including cardiovascular system, and in the generalized arterial calcification of infancy (GACI) [16,26]. The exact molecular mechanisms of tissue calcification associated with aberrant forms of ABCC6 are yet to be determined. However, it has been suggested that ectopic tissue calcification may be caused by aberrant metabolism of inorganic pyrophosphate (PPi), an inhibitor of hydroxyapatite crystal growth, whereas the ABCC6-executed secretion of ATP by the liver, which leads to the rapid conversion of secreted ATP into AMP and PPi, is the main source of PPi in plasma [17,27].

Hitherto, most NVs detected in *ABCC6* are located between exons 24–30. The *c.3421C>T* nonsense variant, resulting in the substitution of arginine with the stop codon, p.R1141X, is among the most frequently found recurrent PSVs in *ABCC6* in European populations [18,28]. Furthermore, *c.3421C>T* in *ABCC6* plays a role in vascular calcification in chronic kidney disease (CKD) [19,29], and its association in heterozygous state with ischemic heart disease has been demonstrated in some case–control studies [20,21,30,31] (though it was not confirmed in further meta-analysis study) [22,32]. The variant *c.4015C>T* (p.R1339C) in the *ABCC6* is also well-known and it has been found previously in several independent studies in patients with PXE [18,28].

In our study, these variants were found in a compound heterozygous state in a patient with AC. Their presence in our patient once again confirms the role of aberrant forms of *ABCC6* in excessive tissue calcification, including AC.

Furthermore, in our presented case, the proband first had the disease after verification of the genetic nature of AC at the age of 1 year and 2 months, and the spectrum of symptoms was dominated by systemic arterial hypertension. There were no signs of ischemia, extracardiac manifestations in the form of angioid streaks on the skin or retinal vascular damage, but this does not exclude the possible appearance of these symptoms during dynamic follow-up. Thus, we acknowledge here that our study lacks long-term follow-up data. It also lacks data from the family screening beyond the results presented in the current report, which might have aided in our understanding of the inheritance patterns and corresponding phenotypes.

Finally, we stress that the approach to the analysis of NGS data varies depending on the patient’s medical history, primarily focusing on identifying causal nucleotide variants in the genes known to be involved in pathogenesis of suspected condition. However, when new clinical information becomes available (for example, in the case described herein, fragmentary calcifications of the branches of several arteries suggesting co-existence in this patient of a condition other than DCM), NGS data can be revisited by a medical geneticist. This may allow additional genetic factors that contribute to the complex phenotype of the patient to be determined. Furthermore, new data in the field of medical genomics accumulates rapidly; for instance, in 2024, the Human Gene Mutations Database expanded with the addition of over 100,000 annotated variants [23,33]. Using NGS of broad gene panels in the clinical setting allows the “revisiting” of previously identified nucleotides based on the most recent literature and data from mutation databases, thus redefining diagnosis.

## 5. Conclusions

The dual presence of different rare hereditary CVDs described herein in one patient, which would not be detected without the NGS of a broad gene panel, underscores the need for comprehensive genetic testing of DCM, particularly in cases with atypical clinical presentation or extracardiac manifestations. We report a novel pathogenic variant *c.542G>T* in the *MYH7* gene with bi-allelic pathogenic nucleotide variants in *ABCC6* in patients with phenotype of DCM and AC. Our findings also reinforce the role of aberrant forms of ABCC6 in pathological tissue calcification, including AC. Bioinformatics reanalysis of NGS data serves as a valuable tool for confirming diagnoses, especially in cases with variable phenotypes.

## Data Availability

Due to privacy protection and ethical restrictions the raw data is not available publically.

## Supplementary Materials

The following supporting information can be downloaded at: https://www.mdpi.com/article/10.3390/ijms26125900/s1.

## Abbreviations

ABC	ATP binding cassette
AC	arterial calcification
CHF	chronic heart failure
DCM	dilated cardiomyopathy
ECG	electrocardiography
EchoCG	echocardiography
EF	ejection fraction
ESC	European Society of Cardiology
GACI	generalized arterial calcification of infancy
HM-ECG	Holter monitoring of ECG
IHD	ischemic heart disease
MSCT	multislice computed tomography
MRP6	Multi-Drug Resistance-Associated Protein-6
MRI	magnetic resonance imaging
NT	proBNP—N-terminal prohormone of brain natriuretic peptide
NV	nucleotide variant
NGS	next-generation sequencing
P/LP	pathogenic/likely pathogenic
PPi	inorganic pyrophosphate
PXE	pseudoxanthoma elasticum
RR	respiratory rate
