# Supplementary material for: A Unique Case of a Child with Two Rare Hereditary Diseases: Familial Dilated Cardiomyopathy and Arterial Calcification
Source: Int J Mol Sci. 2025 Jun 19;26(12):5900. doi: 10.3390/ijms26125900 (PMC12193266; doi:10.3390/ijms26125900)
Supplement: Supplementary file 1 [file ijms-26-05900-s001.zip › ijms-3656452-supplementary.pdf]

**Table S1.** Prediction of variant pathogenicity by in silico tools.

| Tool | Score | Interpretation | Confidence Level |
|---|---|---|---|
| SIFT | 0 | Pathogenic | High |
| Mutation Taster | 1 | Uncertain | Moderate |
| FATHMM | −6.99 | Pathogenic | Moderate |
| DANN | 0.9967 | Uncertain | Moderate |
| M-CAP | 0.9939 | Pathogenic | Moderate |
| REVEL | 0.96 | Pathogenic | Strong |
